# Symptoms of generalized anxiety disorder but not panic disorder at age 15 years increase the risk of depression at 18 years in the Avon Longitudinal Study of Parents and Children (ALSPAC) cohort study

**DOI:** 10.1017/S003329171500149X

**Published:** 2015-08-28

**Authors:** S. J. C. Davies, R. M. Pearson, L. Stapinski, H. Bould, D. M. Christmas, K. S. Button, P. Skapinakis, G. Lewis, J. Evans

**Affiliations:** 1 Centre for Academic Mental Health, School of Social and Community Medicine, University of Bristol, Bristol, UK; 2 Geriatric Psychiatry Division, CAMH, University of Toronto, Toronto, Canada; 3 NHMRC Centre for Research Excellence in Mental Health and Substance Use, National Drug and Alcohol Research Centre, University of New South Wales, Sydney, Australia; 4 Cambridgeshire and Peterborough NHS Foundation Trust, Cambridge, UK; 5 Division of Psychiatry, University College London, London, UK

**Keywords:** Adolescents, anxiety, Avon Longitudinal Study of Parents and Children, depression, generalized anxiety disorder

## Abstract

**Background.:**

Generalized anxiety disorder (GAD) and panic disorder (PD) differ in their biology and co-morbidities. We hypothesized that GAD but not PD symptoms at the age of 15 years are associated with depression diagnosis at 18 years.

**Method.:**

Using longitudinal data from the Avon Longitudinal Study of Parents and Children (ALSPAC) birth cohort we examined relationships of GAD and PD symptoms (measured by the Development and Well-Being Assessment) at 15 years with depression at 18 years (by the Clinical Interview Schedule – Revised) using logistic regression. We excluded adolescents already depressed at 15 years and adjusted for social class, maternal education, birth order, gender, alcohol intake and smoking. We repeated these analyses following multiple imputation for missing data.

**Results.:**

In the sample with complete data (*n* = 2835), high and moderate GAD symptoms in adolescents not depressed at 15 years were associated with increased risk of depression at 18 years both in unadjusted analyses and adjusting for PD symptoms at 15 years and the above potential confounders. The adjusted odds ratio (OR) for depression at 18 years in adolescents with high relative to low GAD scores was 5.2 [95% confidence interval (CI) 3.0–9.1, overall *p* < 0.0001]. There were no associations between PD symptoms and depression at 18 years in any model (high relative to low PD scores, adjusted OR = 1.3, 95% CI 0.3–4.8, overall *p* = 0.737). Missing data imputation strengthened the relationship of GAD symptoms with depression (high relative to low GAD scores, OR = 6.2, 95% CI 3.9–9.9) but those for PD became weaker.

**Conclusions.:**

Symptoms of GAD but not PD at 15 years are associated with depression at 18 years. Clinicians should be aware that adolescents with GAD symptoms may develop depression.

## Introduction

Depression is a leading cause of morbidity with a high cost to society (Sobocki *et al.* 2006). The incidence of depression increases rapidly during late adolescence. For example in the Dunedin study (Jaffee *et al.* 2002) the prevalence rose from 4% at age 15 years to 16% at age 18 years, while in the National Comorbidity Survey adolescent supplement the prevalence of major depression or dysthymia rose from 8.7% at age 13–14 years to 15.7% at age 17–18 years (Merikangas *et al.* 2010). Depression with onset during adolescence carries a high risk of relapse, for example in one study in the USA, 40% of 16 year olds with depression experienced a relapse by the age of 23 years (Lewinsohn *et al*. 1999). In addition, adolescence is an important period for the acquisition of education and development of social and interpersonal skills and a period of depression may disrupt these formative processes with adverse consequences for an individual's future. Understanding the underlying risk factors for depression is therefore important, especially where such risk factors could be identified and modified.

The presence of an anxiety disorder is one such risk factor for the development of depression (Kessler *et al.* 1998; Wittchen *et al.* 2003*a*). Co-morbidity of anxiety disorders and depression is common (Kessler *et al.* 1996, 2005; Wittchen *et al.* 2003*a*, *b*). Kessler *et al.* (1996), using data from the original US National Comorbidity Survey, illustrated that the majority of cases of depression are secondary to another psychiatric diagnosis, with the primary disorder most commonly being an anxiety disorder. Anxiety disorders are often chronic in their course and frequently have onset in childhood or adolescence (Asselmann & Beesdo-Baum, 2015). The general consensus from longitudinal studies in adolescents and in adults is that anxiety disorders precede depression more frequently than vice versa (Pine *et al.* 1998; Stein *et al.* 2001; Goodwin, 2002; Merikangas *et al.* 2003; Bittner *et al.* 2004).

However, diagnostic manuals (World Health Organization, 1993; American Psychiatric Association, 1994) suggest there are several distinct anxiety disorders. While most (e.g. social phobia, specific phobia, agoraphobia and post-traumatic stress disorder) must be linked to well-defined exposures, for generalized anxiety disorder (GAD) and panic disorder this is not the case. The cardinal symptom of GAD is chronic worry whereas a diagnosis of panic disorder requires the experience of recurrent panic attacks. GAD and panic disorder have distinct biology (Wilkinson *et al.* 1998), medical co-morbidities (Davies *et al.* 2012; Davies & Allgulander, 2013) and differ in the frequency of reports of genetic association with depression (Roy *et al.* 1995; Kendler, 1996, Kendler *et al.* 2003, 2007). Factor analyses of cross-sectional data in large populations had suggested that GAD may be more closely associated with depression than is panic disorder (Krueger, 1999; Vollebergh *et al.* 2001; Slade & Watson, 2006). Although this finding was confirmed in a population of adolescents and young adults (Beesdo-Baum *et al.* 2009), there remains a controversy over whether this factor structure is robust to addition of other diagnoses and in other age groups (Wittchen *et al.* 2009).

An analysis from the National Comorbidity Survey Replication reported correlations of 0.62 and 0.48 for 12-month prevalence of major depressive episode with GAD and panic disorder, respectively, in a population-based adult sample (Kessler *et al.* 2005). In a retrospective analysis of longitudinal data, in individuals who had met criteria for both GAD and major depressive episode (Wittchen *et al.* 2003*b*), the depression diagnosis followed that of GAD in 52% of individuals and preceded GAD in only 29%. In a further study which relied on participants’ recall in a population who had experienced depression and panic attacks, a first depressive episode was recalled as occurring after the first panic attack in 43% of cases and preceding it in 31% (Kessler *et al.* 1998). However, in a subsample who had had a diagnosis of panic disorder, which has a higher threshold, 22% recalled that panic disorder preceded the first depressive episode whereas 48% reported that a depressive episode began first.

A limited number of longitudinal studies have attempted to investigate the risk that having an individual anxiety disorder or anxiety symptoms with onset in adolescence carries for subsequent development of depression. Some studies (e.g. Pine *et al.* 1998), having recruited individuals prior to adolescence and only examined anxiety disorder diagnoses specifically relevant to children, do not include disorders well recognized in adulthood such as GAD and panic disorder. However, a study of 2548 individuals aged 14–24 years, each followed up for 4 years (Bittner *et al.* 2004), provides some indication that GAD may have a higher predictive value for depression than does panic disorder. In unadjusted analyses all five of the anxiety disorders examined at baseline in this study, including GAD and panic disorder, conferred an elevated risk of first onset of major depressive disorder. However, after adjustment for prior co-morbid mental disorders, it appeared that panic disorder was no longer associated with subsequent depression. In contrast, an association of GAD with depression remained following adjustment, although there was considerable uncertainty as to the magnitude of the relationship as evidenced by the broad confidence intervals (CIs). Among individuals with GAD or agoraphobia at baseline, co-morbid panic attacks did not increase the risk for major depression. It should be noted, however, that in this study there was considerable heterogeneity of ages, with most of the sample being beyond adolescence even at entry (range 14–24 years). When the link between panic attacks and depression was examined after 10 years of follow-up, panic at baseline did confer an elevated risk for a diagnosis of depression over this longer period (Asselmann *et al.* 2014*a*).

A smaller study of 906 children aged 9–13 years reported no relationship between GAD and subsequent depression at ages 13–19 years (Bittner *et al.* 2007). The authors acknowledged that the predictive value of disorders may differ depending on the developmental stage. They postulated that the restrictive Diagnostic and Statistical Manual of Mental Disorders, 4th edition (DSM-IV) criteria for GAD in children and adolescents (American Psychiatric Association, 1994) made it more difficult to demonstrate associations with other psychiatric disorders. In contrast, GAD's diagnostic predecessor (overanxious disorder) did show an association with depression, as it had done in earlier studies (Pine *et al.* 1998). To our knowledge no study has specifically examined the impact of GAD and panic disorder from a fixed point in adolescence on the prevalence of depression at the onset of adulthood when the rise in incidence of depression is steepest.

While some evidence therefore suggests that GAD may be more closely associated with depression than is panic disorder, a single definitive mechanism that might underlie this link cannot be identified. Possible mechanisms which may contribute include:

(*a*) Genetics, as evidenced by the overlaps between GAD and depression discussed above. This might involve a genetic pleiotropy involving multiple polymorphisms predisposing to both depression and GAD simultaneously, one example being a polymorphism in the gene coding for the serotonin 5HT-1A receptor (Molina *et al.* 2011), although this has not been replicated in genome-wide association studies.
(*b*) A common biological mechanism such as cortisol dysregulation which is known to be involved in the aetiology of depression. A recent paper examining evidence of hypothalamic–pituitary–adrenal axis dysfunction across the anxiety disorders found evidence in GAD, but was inconclusive for panic disorder (Abelson *et al.* 2007; Faravelli *et al.* 2012).
(*c*) A vulnerability of GAD and depression, but not panic disorder to a specific but as yet unidentified risk factor (although a risk factor answering this description was not identified in an earlier examination of risk factors for depression and individual anxiety disorders; Beesdo *et al.* 2010).
(*d*) A psychological effect of one disorder leading to the other (e.g. generalized anxiety causing demoralization and leading to depression).
(*e*) GAD but not panic disorder being an early developmental manifestation of depression.

The Avon Longitudinal Study of Parents and Children (ALSPAC; Golding *et al.* 2001) provides an opportunity to study factors contributing to increased risk of depression at 18 years in a prospective cohort study design. In the present study we will consider symptoms of anxiety disorders in adolescence as risk factors for the subsequent development of depression by the age of 18 years, making the distinction between symptom patterns suggestive of GAD and panic disorder. Despite clear-cut differences in symptoms and diagnostic criteria, few studies have attempted to distinguish between the predictive ability of these two common forms of anxiety for the risk of future depression, which is important to know for the detection of depression and potentially for the prevention of its onset. Given the genetic associations between GAD and depression, and the suggestion of an association of these disorders at 4 years in an earlier longitudinal study, we hypothesized that GAD but not panic disorder symptoms at age 15 years are associated with a diagnosis of depression at 18 years. Elucidating this relationship may have practical implications for the prevention of depression.

## Method

### Data source

The sample comprised participants from the ALSPAC. All pregnant women resident in the former Avon Health Authority in south-west England having an estimated date of delivery between 1 April 1991 and 31 December 1992 were invited to take part. The children of 15 247 pregnancies were recruited. Compared with the 1991 National Census data of residents in Avon, the ALSPAC had a slightly greater proportion of mothers who were married or cohabiting and who were owner-occupiers but was similar for other demographic variables. Ethical approval for the study was obtained from the ALSPAC Law and Ethics Committee and the Local Research Ethics Committees. More detailed information on the ALSPAC is available on the study website which contains details of all the data that are available through a fully searchable data dictionary (http://www.bris.ac.uk/alspac/researchers/data-access/data-dictionary/). Detailed information has been collected on the cohort since early pregnancy, including regular self-reported information from mothers and children and face-to-face assessments in research clinics (Boyd *et al.* 2013). The current study uses data from the remaining sample of ALSPAC offspring who attended the two most recent research clinics for the children at age 15 and 18 years.

### Sample size

Our starting sample was those with complete anxiety and depression data from the Development and Well-Being Assessment (DAWBA; Goodman *et al.* 2000), the exposure variable, at age 15 years (*n* = 5365). Those in the highest of the six bands of symptoms for depression on the DAWBA (indicating a probable diagnosis of depression based on symptoms over the past 2 weeks) at 15 years were removed from the sample at the outset (*n* = 89), including 40 out of 269 (15%) of those with the highest levels of GAD and four out of 32 (13%) of those with the highest levels of panic symptoms. Of the remaining sample, 3635 adolescents also had complete depression outcome data using the Clinical Interview Schedule – Revised (CIS-R) at the age of 18 years. A final sample with complete data across all exposure, outcome and confounding variables comprised 2835 participants. However, all missing data were imputed and all analyses described below were repeated using the same starting sample size (*n* = 5365) in final sensitivity analyses.

We imputed for missing data because missing data can bias results if the data are not missing completely at random. Given that there is substantial information on sociodemographic variables in the ALSPAC that predict missingness, missing information can be assumed dependent on observed data. Thus we employed a fully conditional specification as implemented in the Multivariate Imputation by Chained Equations (MICE) algorithm in STATA 12, using all variables described in the analyses and additional auxiliary variables predictive of incomplete variables and/or missingness (these included sociodemographic indicators and earlier continuous measures of depression; full list available on request) to predict missing data across 100 imputed datasets. The resulting Monte Carlo errors were less than 10% of the standard error and fraction of missing information (FMI) values were no larger than 0.4, indicating that 100 imputed datasets were sufficient. Analyses were conducted post-imputation by combining estimates across imputed datasets using Rubin's rules.

### Measures

#### GAD and panic at age 15 years

Symptoms of GAD, panic and depression were measured by the DAWBA at the age of 15 years. We used the pencil and paper version of the DAWBA for self-completion. The DAWBA consists of questions about child mental health symptoms and their impact. The questions for each disorder follow the diagnostic criteria operationalized in the DSM-IV (American Psychiatric Association, 1994) or the International Classification of Diseases, 10th revision (ICD-10) Diagnostic Criteria for Research (World Health Organization, 1993). Accordingly, questions on GAD refer to the last 6 months and those on panic disorder to the last 4 weeks. Note that for GAD, the DAWBA employs the diagnostic criteria F93.80 from ICD-10's Diagnostic Criteria for Research, termed ‘generalized anxiety disorder of childhood’ which closely resembles the criteria for GAD in the DSM-IV (category 300.02) but differs markedly from ICD-10's adult GAD criteria (F41.1) which require the presence of autonomic symptoms and/or dry mouth. Each section contains 20–25 questions. Ordered categorical variables from 1 to 6 or ‘bands’ for each ICD-10 or DSM-IV disorder are derived from symptoms using a computerized algorithm (Goodman *et al.* 2011). This approach has the advantage of describing the symptom intensity for disorders along a continuum which may be particularly helpful in adolescence, where some disorders are rare but the presence of symptoms which do not meet the threshold for diagnosis is much more common. The levels of symptoms for each ‘band’ were chosen to provide an approximately evenly spaced progression in terms of the log odds of the child having the disorder in question (Goodman *et al.* 2011). Binary indicators of probable diagnosis are also generated. However, we used ordered-categorical measures in order to make full use of the symptom variation and to allow investigation of dose–response effects. Due to low frequency within some bands, we collapsed data from the original six levels to form three ordinal levels ranging from the lowest to highest probability of having the disorder in question (i.e. low, medium and high symptom bands). In addition, continuous symptom scores for GAD and panic were derived from the sum of all symptom items within the relevant section of the DAWBA. These symptom scores were used as exposure variables in secondary analyses.

#### Depression at age 18 years (outcome variable)

Depression at 18 years old was measured using the computerized version of the CIS-R. The CIS-R is a computerized interview that derives a diagnosis of depression according to ICD-10 criteria (World Health Organization, 1993), the time-frame being the past 2 weeks. The interview has been fully standardized and is equally reliable whether conducted by a lay or clinically trained interviewer or self-administered on the computerized version (Lewis, 1994). The CIS-R is designed for, and has been widely used within, community samples including the National Surveys of Psychiatric Morbidity and the 1958 birth cohort. A binary variable indicating presence *v*. absence of a diagnosis of depression was taken as the outcome measure.

#### Confounding variables

Variables that are associated with both anxiety and depression in adolescence were selected. These included maternal education (Letourneau *et al.* 2006; Chen & Li, 2009; Park *et al.* 2013), social class (using the ‘Standard Occupational Classification’ of the Office of Population Censuses and Surveys, 1995), child gender, birth order (Gates *et al.* 1988), child smoking status (ever smoked a cigarette, yes/no) and frequency of child alcohol use (six-level ordinal variable from never to daily alcohol consumption).

### Analyses

The exposure variable was DAWBA symptom bands at age 15 years (three-level ordered categorical variable) and the outcome was diagnosis of depression at age 18 years (binary variable). The associations between GAD and panic symptom bands and depression diagnosis were first investigated within separate logistic regression models using STATA 12 (StataCorp, 2011). We then investigated the association with both these exposures in the same logistic regression model. Confounding variables were then introduced into the models, including adjustments for depression symptoms at age 15 years (ordered categorical variable, using DAWBA bands from 1–5 with those in band 6 having been excluded). These analyses were repeated using symptom scores for GAD and panic as continuous exposure variables. Finally, the models described above were repeated following imputation for missing data. The population attributable fraction (PAF) was calculated as (1 – population unattributable fraction) using the ‘punaf’ command in STATA. This calculates the population unattributable fraction as the ratio of the log of scenario means for the outcome (depression at 18 years) in the baseline scenario *v*. the ‘ideal’ scenario in which the risk factor (here being medium or high anxiety at 15 years) is set to zero.

## Results

### Descriptive statistics

Sample demographics for the adolescents who comprised our complete case sample as compared with the rest of the ALSPAC are given in Table 1. As can be seen, the adolescents with complete case data came from more socially advantaged families.

**Table 1.** Sample demographics

	ALSPAC sample lacking exposure data, *n* (%)	Exposure (DAWBA) data available, *n* (%)	Complete cases and depressed cases at 15 years removed, *n* (%)
Participants, *n*	9613	5365	2835
Female	6570 (45)	2820 (53)	1557 (55)
Maternal education			
Up to 16 years only	4808 (70)	2501 (52)	1347 (48)
Up to 18 years	1402 (20)	1402 (29)	889 (31)
University degree	723 (10)	887 (19)	599 (21)
Social class			
Highest: 1	263 (5)	334 (8)	250 (9)
2	1604 (28)	1581 (36)	1066 (38)
3	3060 (53)	2060 (47)	1290 (46)
Lowest: 4 or 5	838 (14)	385 (9)	228 (8)
Mean age of mother, years (s.d.)	26.7 (5)	28.5 (5)	28.9 (4)
First born	4736 (58)	2518 (51)	1353 (48)
Never smoked (measured at age 15 years)	No data available	2778 (51.8)	1613 (56.9)
Alcohol consumption	No data available		
Never		754 (14.1)	424 (14.9)
Once/twice		604 (11.3)	330 (11.6)
Used to drink but not now		166 (3.1)	79 (2.8)
Sometimes but less than once a week		2666 (49.8)	1458 (51.4)
1–2 times a week		923 (17.2)	441 (15.6)
Drink more than twice a week		207 (3.9)	92 (3.2)
Drink every day		33 (0.6)	12 (0.4)

ALSPAC, Avon Longitudinal Study of Parents and Children; DAWBA, Development and Well-Being Assessment; s.d., standard deviation.

### Co-morbidity

Frequency of scoring within each of the DAWBA bands, co-morbidity and symptom scores are given in Tables 2 and 3. As can be seen, a large proportion of those with high levels of panic have high levels of GAD.

**Table 2.** Mean total GAD symptom score according to each DAWBA symptom intensity band^a^ for GAD for the whole sample at age 15 years excluding those depressed (*n* = 5276)

	Frequency, *n* (%)	Mean GAD symptom score (s.d.)	Co-morbidity in high panic symptom band, *n* (%)
Low GAD symptom band	2096 (40)	0.9 (0.8)	1 (0.1)
Medium GAD symptom band	2951 (56)	2.8 (1.6)	18 (0.6)
High GAD symptom band	229 (4)	17.8 (4.4)	9 (4)

GAD, Generalized anxiety disorder; DAWBA, Development and Well-Being Assessment, s.d., standard deviation.

a GAD measured by the DAWBA and divided into low, medium and high symptom bands (see the Method section).

**Table 3.** Mean total panic symptom score according to each DAWBA symptom intensity band^a^ for panic for the whole sample at age 15 years excluding those depressed (*n* = 5276)

	Frequency, *n* (%)	Mean panic symptom score (s.d.)	Co-morbidity in high GAD symptom band, *n* (%)
Low panic symptom band	5140 (97)	0.005 (0.2)	199 (0.04)
Medium panic symptom band	108 (2)	9.4 (3.1)	21 (19)
High panic symptom band	28 (1)	11.5 (3.3)	9 (32)

DAWBA, Development and Well-Being Assessment, s.d., standard deviation; GAD, generalized anxiety disorder.

a Panic symptom intensity measured by the DAWBA and divided into low, medium and high symptom bands (see the Method section).

### Association between GAD and panic with confounding variables

After removing those with co-morbid depression, those in higher GAD symptom bands at age 15 years were more likely to be girls (χ^2^_2_ = 182, *p* < 0.001: 75% of adolescents in the highest band were girls), to drink alcohol more frequently (χ^2^_12_ = 29, *p* < 0.001) and to have ever smoked a cigarette (χ^2^_2_ = 14, *p* < 0.001). A similar pattern was seen for panic: those in higher panic symptom bands were more likely to be girls (χ^2^_2_ = 27, *p* < 0.001: 75% of adolescents in the highest band were girls), to drink alcohol more frequently (χ^2^_12_ = 31, *p* < 0.001) and to have ever smoked a cigarette (χ^2^_2_ = 5, *p* < 0.084, 68% of those in the top band compared with only 47% in the lowest band had tried a cigarette). There was no association between either GAD or panic and maternal education, social class or parity.

### Main analyses

As can be seen in Table 4, adolescents with medium and high levels of either GAD or panic symptoms at the age of 15 years more frequently received a diagnosis of depression at 18 years. Univariate logistic regression models (Tables 5 and 6) provide strong evidence for an association between GAD and depression [odds ratio (OR) for high GAD symptom band *v*. low GAD symptom band = 6.6, 95% CI 3.9–11.2] and a trend towards an association between panic and depression at age 18 years (OR for high panic symptom band *v*. low panic symptoms band = 3.1, 95% CI 0.9–10.8). The association of high GAD symptoms with depression was robust to the addition of panic (model 2) and to the addition of the six confounding variables described above (model 3, OR for high GAD symptom band *v*. low GAD symptom band = 5.2, 95% CI 3.0–9.1) as well as the addition of baseline depression (model 4, OR for high GAD symptom band *v*. low GAD symptom band = 3.8, 95% CI 2.1–6.7). In contrast, the association with panic diminished markedly once GAD was included in the model (OR for high panic symptom band *v*. low panic symptom band = 1.6, 95% CI 0.4–5.9) and remained so after adjustment for the further confounders and baseline depression. This reflects the relatively large proportion of those with high panic symptom scores also having high GAD symptom scores (32%, see above). This pattern of results was also found when using the symptom scores as continuous exposure variables (Tables 5 and 6). The association between GAD and depression was strengthened following imputation for missing data, whilst any association with panic was further weakened (see Tables 7 and 8).

**Table 4.** Frequencies of depression^a^ at 18 years according to GAD and panic symptom severity^b^ at 15 years by DAWBA symptom bands, for the non-depressed sample at 15 years (*n* = 3635)

	Proportion (%) of those in each symptom band at 15 years who go on to have depression^a^ at 18 years
Low GAD symptom band	54/1433 (4)
Medium GAD symptom band	163/ 1876 (8)
High GAD symptom band	38/124 (23)
Low panic symptom band	240/3544 (7)
Medium panic symptom band	12/72 (17)
High panic symptom band	3/16 (17)

GAD, Generalized anxiety disorder; DAWBA, Development and Well-Being Assessment; CIS-R, Clinical Interview Schedule – Revised.

a Depression identified by the CIS-R.

b GAD and panic symptom intensity measured by the DAWBA and divided into low, medium and high symptom bands (see the Method section).

**Table 5.** OR for depression at 18 years according to GAD symptom band at age 15 years (compared with the lowest symptom band) and continuous symptom score in separate logistic regression models, combined models and following adjustments for confounding variables (models are for complete cases across all exposure, outcome and confounding variables with exclusion of those with depression at 15 years; *n* = 2835)

	Model 1: risk for depression^a^ at 18 years, unadjusted		Model 2: risk for depression^a^ at 18 years, adjusted for panic		Model 3: risk for depression^a^ at 18 years, adjusted for panic^b^ and confounders^c^		Model 4: risk for depression^a^ at 18 years, adjusted for panic^b^ and confounders^c^ + depression symptom bands^d^ at 15 years	
									OR (95% CI)	*p*	OR (95% CI)	*p*	OR (95% CI)	*p*	OR (95% CI)	*p*
ORs for depression at 18 years with GAD symptom intensity^b^ at 15 years as exposure								
Low GAD symptom band	1 (reference category)		1 (reference category)		1 (reference category)		1 (reference category)	
Medium GAD symptom band	1.9 (1.3–2.7)		1.9 (1.3–2.7)		1.7 (1.2–2.5)		1.5 (0.9–2.1)	
High GAD symptom band	6.6 (3.9–11.2)		6.3 (3.7–10.8)		5.2 (3.0–9.1)		3.8 (2.1–6.7)	
Overall effect using likelihood ratio test		< 0.0001		< 0.0001		< 0.0001		0.0001
Continuous GAD symptom score^e^	1.5 (1.4–1.7)	< 0.001	1.5 (1.4–1.7)	< 0.001	1.5 (1.3–1.7)	< 0.001	1.4 (1.2–1.6)	< 0.001

OR, Odds ratio; GAD, generalized anxiety disorder; CI, confidence interval; CIS-R, Clinical Interview Schedule – Revised; DAWBA, Development and Well-Being Assessment.

a Depression identified by the CIS-R.

b GAD and panic symptom intensity at 15 years measured by the DAWBA and divided into low, medium and high symptom bands (see the Method section).

c Social class, maternal education, birth order, child gender, child smoking status (ever smoked a cigarette, yes/no), child frequency of alcoholic drinks (six levels, ‘I have never tried alcohol’ up to ‘I drink every day’).

d Depression symptom intensity at 15 years measured by the DAWBA, divided into bands 1–5 (as described in the Method section, those in band 6 at age 15 years were excluded).

^e^OR reflects increased odds for a 1 standard deviation symptom increase.

**Table 6.** OR for depression at 18 years according to panic symptom severity at age 15 years (compared with the lowest symptom band) and continuous symptom score in separate logistic regression models, combined models and following adjustments for confounding variables (models are for complete cases across all exposure, outcome and confounding variables with exclusion of those with depression at 15 years; *n* = 2835)

	Model 1: risk for depression^a^ at 18 years, unadjusted		Model 2: risk for depression^a^ at 18 years, adjusted for GAD		Model 3: risk for depression^a^ at 18 years, adjusted for GAD^b^ and confounders^c^		Model 4: risk for depression^a^ at 18 years, adjusted for GAD^b^ and confounders^c^ + depression symptom bands^d^ at 15 years	
	OR (95% CI)	*p*	OR (95% CI)	*p*	OR (95% CI)	*p*	OR (95% CI)	*p*
ORs for depression at 18 years with panic symptom intensity^b^ at 15 years as exposure								
Low panic symptom band	1 (reference category)		1 (reference category)		1 (reference category)		1 (reference category)	
Medium panic symptom band	2.1 (0.9–5.0)		1.4 (0.6–3.5)		1.4 (0.6–3.5)		1.3 (0.5–3.2)	
High panic symptom band	3.1 (0.9–10.8)		1.6 (0.4–5.9)		1.3 (0.3–4.8)		1.2 (0.3–4.5)	
Overall effect using likelihood ratio test		0.094		0.629		0.737		0.838
Continuous symptom score^e^	1.1 (1.0–1.1)	0.006	1.0 (1.0–1.0)	0.530	1.0 (1.0–1.0)	0.744	1.0 (1.0–1.0)	0.790

OR, Odds ratio; GAD, generalized anxiety disorder; CI, confidence interval; CIS-R, Clinical Interview Schedule – Revised; DAWBA, Development and Well-Being Assessment.

a Depression identified by the CIS-R.

b GAD and panic symptom intensity at 15 years measured by the DAWBA and divided into low, medium and high symptom bands (see the Method section).

c Social class, maternal education, birth order, child gender, child smoking status (ever smoked a cigarette, yes/no), child frequency of alcoholic drinks (six levels, ‘I have never tried alcohol’ up to ‘I drink every day’).

d Depression symptom intensity at 15 years measured by the DAWBA, divided into bands 1–5 (as described in the Method section, those in band 6 at age 15 years were excluded).

^e^OR reflects increased odds for a 1 standard deviation symptom increase.

**Table 7.** Main analyses: model 3 before and after imputing missing data, according to GAD symptom band

	Risk for depression^a^ at 18 years, adjusted for panic^b^ and confounders^c^, complete cases (*n* = 2835)		Risk for depression^a^ at 18 years, adjusted for panic^b^ and confounders^c^, after imputing for missing data (*n* = 5276)	
	OR (95% CI)	*p*	OR (95% CI)	*p*
Low GAD symptom band^b^	1 (reference category)		1 (reference category)	
Medium GAD symptom band^b^	1.7 (1.2–2.5)		2.0 (1.–2.7)	
High GAD symptom band^b^	5.2 (3.0–9.1)		5.8 (3.7–9.2)	
Continuous GAD symptom score^d^	1.3 (1.2–1.4)	< 0.001	1.3 (1.2–1.4)	< 0.001

OR, Odds ratio; CI, confidence interval; GAD, generalized anxiety disorder; CIS-R, Clinical Interview Schedule – Revised; DAWBA, Development and Well-Being Assessment.

a Depression identified by the CIS-R.

b GAD and panic symptom intensity at 15 years measured by the DAWBA and divided into low, medium and high symptom bands (see the Method section).

c Social class, maternal education, birth order, child gender, child smoking status (ever smoked a cigarette, yes/no), child frequency of alcoholic drinks (six levels, I have never tried alcohol up to I drink every day).

^d^OR reflects increased odds for a 1 standard deviation symptom increase.

**Table 8.** Main analyses: model 3 before and after imputing missing data, according to panic symptom band

	Risk for depression^a^ at 18 years, adjusted for GAD^b^ and confounders^c^, complete cases (*n* = 2835)		Risk for depression^a^ at 18 years, adjusted for GAD^b^ and confounders^c^, after imputing for missing data (*n* = 5276)	
	OR (95% CI)	*p*	OR (95% CI)	*p*
Low panic symptom band^b^	1 (reference category)		1 (reference category)	
Medium panic symptom band^b^	1.4 (0.6–3.5)		1.5 (0.8–2.8)	
High panic symptom band^b^	1.3 (0.3–4.8)		1.1 (0.3–4.1)	
Continuous GAD symptom score^d^	1.0 (0.9–1.1)	0.760	1.0 (0.9–1.0)	0.402

OR, Odds ratio; CI, confidence interval; GAD, generalized anxiety disorder; CIS-R, Clinical Interview Schedule – Revised; DAWBA, Development and Well-Being Assessment.

a Depression identified by the CIS-R.

b GAD and panic symptom intensity at 15 years measured by the DAWBA and divided into low, medium and high symptom bands (see the Method section).

c Social class, maternal education, birth order, child gender, child smoking status (ever smoked a cigarette, yes/no), child frequency of alcoholic drinks (six levels, I have never tried alcohol up to I drink every day).

d OR reflects increased odds for a 1 standard deviation symptom increase.

The PAF provides an index of the proportion of depression cases at 18 years that could be prevented by removing moderate or high symptoms of GAD at 15 years (assuming a causal relationship). Based on the fully adjusted regression (model 4) described above the PAF is 0.37 (0.20–0.50), suggesting that, if causal, successfully eliminating moderate or high symptoms of GAD at 15 years would prevent 37% of depressed cases at 18 years.

## Discussion

Our findings confirm our original hypotheses that symptoms of GAD but not panic disorder at the age of 15 years are associated with depression at 18 years. Symptoms of GAD at 15 years are strongly associated with subsequent depression, and this relationship remained after adjustment for confounders including baseline depressive symptoms and was strengthened further on missing data imputation. The elevated risk for depression at 18 years applied even to those whose GAD symptoms at 15 years were rated as moderate. However, the apparent association between panic symptoms and later depression on the unadjusted analysis was a result of confounding by an association between panic and GAD. Overall, the present study strengthens the existing literature suggesting that depression is more closely associated with GAD than with panic disorder. It supports the broader concept that GAD and panic disorder differ in their co-morbidities.

We note that since lifetime incidence of depression prior to the age of 15 years was not assessed, these data do not allow us to say with certainty that GAD invariably precedes depression since it is conceivable that some participants had experienced depression earlier in their development which had remitted some time before their DAWBA at age 15 years. However given the relatively low prevalence of depression at 15 years we believe that such individuals are likely to be few in number. Despite an earlier analysis failing to identify temperament, personality or environmental risk factors shared by GAD and depression but not other anxiety disorder (Beesdo *et al.* 2010), there remains the possibility that our results reflect a developmental phenomenon, in that the expression of GAD symptoms could represent an early manifestation of depression if the two symptom groups had common aetiology through shared genetic, biological or environmental factors, or were manifestations of the same disorder. In some individuals the brain at 15 years may be more readily able to express anxiety, worry and the somatic symptoms of GAD than sadness, misery or the biological symptoms of depression despite the aetiology being the same. Thus development of GAD symptoms at 15 years could be seen as evidence of an individual being on a trajectory due to common underlying causes which will result in depression at 18 years unless adaptation occurs or treatment is instigated. However, some authors have argued that depression and GAD have sufficient differences in terms of risk factors, co-morbidities and treatment strategies that it is unrealistic to consider them as manifestations of a single underlying cause (Hettema, 2008). As such, it remains possible that the presence of generalized anxiety with pervasive worry causes demoralization which is more likely to lead to depression as a separate disorder, than is the more episodic phenomenon of panic attacks experienced in panic disorder.

Whether or not GAD and depression are truly separate disorders (Mennin *et al.* 2008), our study suggests that late adolescence is a period where GAD symptoms are common and are linked to an increased prevalence of depression subsequently. It is important to recognize the developmental context: the period from age 15 to 18 years is when the sharpest rise in the incidence of depression is observed, a disorder that has huge public health importance. Therefore it is possible that this period may provide an opportunity to identify and treat symptoms of GAD which is beneficial in itself and may have the added benefit of reducing the risk of a subsequent depressive disorder occurring. Identification and treatment of panic disorder remains important as this disorder is unpleasant and disabling in its own right. The lack of any association between panic symptoms and subsequent depression contrasts with the reported co-morbidity of panic disorder and depression in the general population (Skapinakis *et al.* 2011), suggesting that these are separate disorders that may co-occur without having a developmental link. However, it remains possible that depression following on from panic merely takes longer to emerge as suggested by reports of an association after 10 years’ follow-up (Asselmann *et al.* 2014*a*) but not after 4 years (Bittner *et al.* 2004). Indeed, treatment of panic attacks in adolescents and young adults appeared to reduce the incidence of depression at the 10-year follow-up time point (Asselmann *et al.* 2014*b*).

GAD can be treated successfully, both by psychological therapies (Hunot *et al.* 2007) and by medications (Ravindran & Stein, 2010; Baldwin *et al.* 2014). While the evidence is more limited for some drug classes in children than is the case in adults, cognitive–behavioural therapy is effective both in children and adolescents (Compton *et al.* 2004; Ishikawa *et al.* 2007). We hypothesize that successful treatment of adolescents with GAD could have the additional beneficial effect of reducing the risk of developing depression and its many undesirable consequences. Such an intervention could be considered to be a form of indicated prevention (Mrazek 2& Haggerty, 1994). The impact of such an indicated prevention would need to be evaluated and balanced against any possible risk of self-harm (Olfson *et al.* 2003; Hammad, 2004).

We advocate better mechanisms for identifying adolescents who experience GAD, including its core symptom of chronic worry. It is known that recognition rates for anxiety disorders in primary care are suboptimal (Wittchen *et al.* 2002, 2012). Although administration of screening instruments for anxiety disorders has been shown to be feasible in adults (Wittchen *et al.* 2002) and children (Simon & Bögels, 2009) it is unclear whether it could be justified in primary care populations. A systematic review examining the value of screening for depression in primary care using a routinely administered questionnaire concluded that the benefits were marginal (Gilbody *et al.* 2005) although it was acknowledged that a more sophisticated two-stage procedure for screening and case finding had yet to be evaluated.

The strengths of the study are the large sample size, that it is population-based, with both exposure and outcome data being collected from individuals at fixed ages, and the longitudinal design. Possible limitations include the attrition rates in the ALSPAC reducing representativeness and applicability of the sample to the general population. This problem was addressed at least in part by use of imputation techniques for missing data. While as many as 46% of individuals had one or more missing data value, the benefits of imputation are that biases associated with exclusion of subjects who do not provide complete data are avoided. As there was little difference between imputed and non-imputed results, our confidence in our findings is increased. The number of participants in the highest DAWBA symptom band for panic was small. The smaller number of participants in this symptom band reduces the power to find an association with depression at 18 years. The distribution of the panic symptom bands at 15 years reflects the fact that the panic disorder diagnosis is relatively rare at that age, but panic attacks are more common and our approach of using the DAWBA symptom bands as exposure variables rather than simply the presence or absence of a full clinical diagnosis mitigates the problem to some extent. Finally, the DAWBA estimates likelihood of diagnosis of GAD based on DSM-IV and the ICD-10 diagnostic criteria for research category F93.80. In both cases these criteria include symptoms such as sleep disturbance and poor concentration, which are also considered features of depression. Thus, using the DAWBA may tend to increase the overlap of GAD and depression compared with any instrument which used ICD's more autonomically oriented F41.1 criteria.

Further studies are required to establish whether identifying and treating GAD in adolescents reduces either the incidence or the severity of subsequent depression.
